# Pretransplant IgA-Anti-Beta 2 Glycoprotein I Antibodies As a Predictor of Early Graft Thrombosis after Renal Transplantation in the Clinical Practice: A Multicenter and Prospective Study

**DOI:** 10.3389/fimmu.2018.00468

**Published:** 2018-03-12

**Authors:** Jose M. Morales, Manuel Serrano, Jose Angel Martinez-Flores, Fracisco Javier Gainza, Roberto Marcen, Manuel Arias, Fernando Escuin, Dolores Pérez, Amado Andres, Miguel Angel Martínez, Naroa Maruri, Eva Alvarez, José Luis Castañer, Marcos López-Hoyos, Antonio Serrano

**Affiliations:** ^1^Hospital 12 de Octubre, Nephrology Department, Healthcare Research Institute (Imas12), Madrid, Spain; ^2^Hospital 12 de Octubre, Immunology Department, Healthcare Research Institute (Imas12), Madrid, Spain; ^3^Nephrology Department, Hospital Universitario de Cruces, Biocruces Health Research Institute, Baracaldo, Spain; ^4^Hospital Ramon y Cajal, Nephrology Department, Madrid, Spain; ^5^Hospital Ramon y Cajal, Immunology Department, Madrid, Spain; ^6^Hospital Marques de Valdecilla, Nephrology Department, Santander, Spain; ^7^Hospital Marques de Valdecilla, Immunology Department, Santander, Spain; ^8^Hospital la Paz, Nephrology Department, Madrid, Spain

**Keywords:** graft thrombosis, kidney transplant, autoimmunity, autoantibodies, antiphospholipid syndrome, antiphospholipid antibodies, B2GP1, IgA

## Abstract

**Background:**

Graft thrombosis is a devastating complication after renal transplantation. We recently described the association of anti-beta-2-glycoprotein-I (IgA-ab2GP1) antibodies with early graft loss mainly caused by thrombosis in a monocenter study.

**Methods:**

Multicenter prospective observational cohort study.

**Setting and participants:**

Seven hundred forty patients from five hospitals of the Spanish Forum Renal Group transplanted from 2000 to 2002 were prospectively followed-up for 10 years.

**Outcomes:**

Early graft loss and graft loss by thrombosis.

**Measurements:**

The presence of IgA anti-B2GP1 antibodies in pretransplant serum was examined using the same methodology in all the patients.

**Results:**

At transplantation, 288 patients were positive for IgA-B2GP1 (39%, Group-1) and the remaining were negative (Group-2). Graft loss at 6 months was higher in Group-1 (12.5 vs. 4.2% *p* < 0.001), vessel thrombosis being the most frequent cause of early graft loss, especially in Group-1 (6.9 vs. 0.4% *p* < 0.001). IgA-aB2GP1 was the most important independent risk factor for graft thrombosis (hazard ratio: 13.83; 95% CI: 3.17–60.27, *p* < 0.001). Furthermore, the, presence of IgA-aB2GP1 was associated with early graft loss and delayed graft function. At 10 years, survival figures were also lower in Group-1: graft survival was lower compared with Group-2 (60.4 vs. 76.8%, *p* < 0.001). Mortality was significantly higher in Group-1 (19.8 vs. 12.2%, *p* = 0.005).

**Limitations:**

Patients were obtained during a 3-year period (1 January 2000–31 December 2002) and kidneys were only transplanted from brain-dead donors. Nowadays, the patients are older and the percentage of sensitized and retransplants is high.

**Conclusion:**

In a prospective observational multicenter study, we were able to corroborate that pretransplant presence of IgA-aB2GP1 was the main risk factor for graft thrombosis and early graft loss. Therefore, a prospective study is needed to evaluate the efficacy and safety of prophylactic anticoagulation to avoid this severe complication.

## Introduction

The introduction of modern immunosuppression, advances in the control of infections, better methodologies, and improvement in histocompatibility tests in recent decades have substantially increased short- and long-term results after renal transplantation. However, the percentage of patients who suffer graft loss in the first months posttransplant (mainly by thrombosis) has remained unchanged (5–8%) (1).

Humoral immune response to the allograft after kidney transplantation is one of the main factors responsible for the deterioration of graft function, or even graft loss. The main target of this immune attack is the donor major histocompatibility complex (alloreactivity) (2). It has been recently described that antibody-based autoimmune responses may also affect the outcome of renal transplantation (3). The main antigens associated with an autoimmune-mediated allograft response are angiotensin 1 receptor (4), endotelin 1 receptor (5), LG3 fragment of perlecan (6), and β_2_ glycoprotein I (B2GP1) (7).

Antiphospholipid antibodies (aPL) are a group of autoantibodies directed against phospholipid-binding plasma proteins, including both those circulating in the blood and/or located in the plasma membrane of blood vessel cells. Antiphospholipid syndrome (APS) is an autoimmune disorder characterized by the presence of circulating aPL associated with vessels thrombosis or adverse pregnancy outcome (8).

B2GP1 is a protein that is mainly synthesized not only in the liver but also in kidney and heart. It is localized in plasma and in the membrane of endothelial cells, the platelets being the most frequent antigen recognized by pathogenic aPL (9, 10). The prevalence of anti-B2GP1 antibodies of IgA isotype (IgA-aB2GP1) is higher in patients with chronic kidney disease than in the general population (30 vs. 1.5%) (11), and an association between these antibodies in patients undergoing hemodialysis and thrombotic events and mortality has been found (12, 13).

We recently described the association of a variety of IgA anti-beta-2 glycoprotein-I (aB2GP1) antibodies with early loss of kidney grafts, mainly from thrombosis (7). This observation was confirmed in a large historical cohort of patients transplanted over a 12-year period in a single hospital (14).

Our work has aimed to corroborate the association of IgA aB2GP1 with early graft thrombosis after renal transplantation in a prospective multicenter study.

## Materials and Methods

### Study Design

We performed an observational, “non-interventional” follow-up study that included patients transplanted in five hospitals of the Spanish Forum Renal Group cohort. The original series (“Forum Renal”) included all transplanted patients, without exclusions, during 2000–2002 in 14 renal transplant units in Spain (*N* = 2,600).

In our study, only patients from the five units with stored pretransplant serum samples were included, it not being possible to include the patients from the remaining centers because pretransplant serum samples were not available. Patients with hemolytic uremic syndrome, factor V Leiden, or primary APS were excluded.

**Figure 1:**
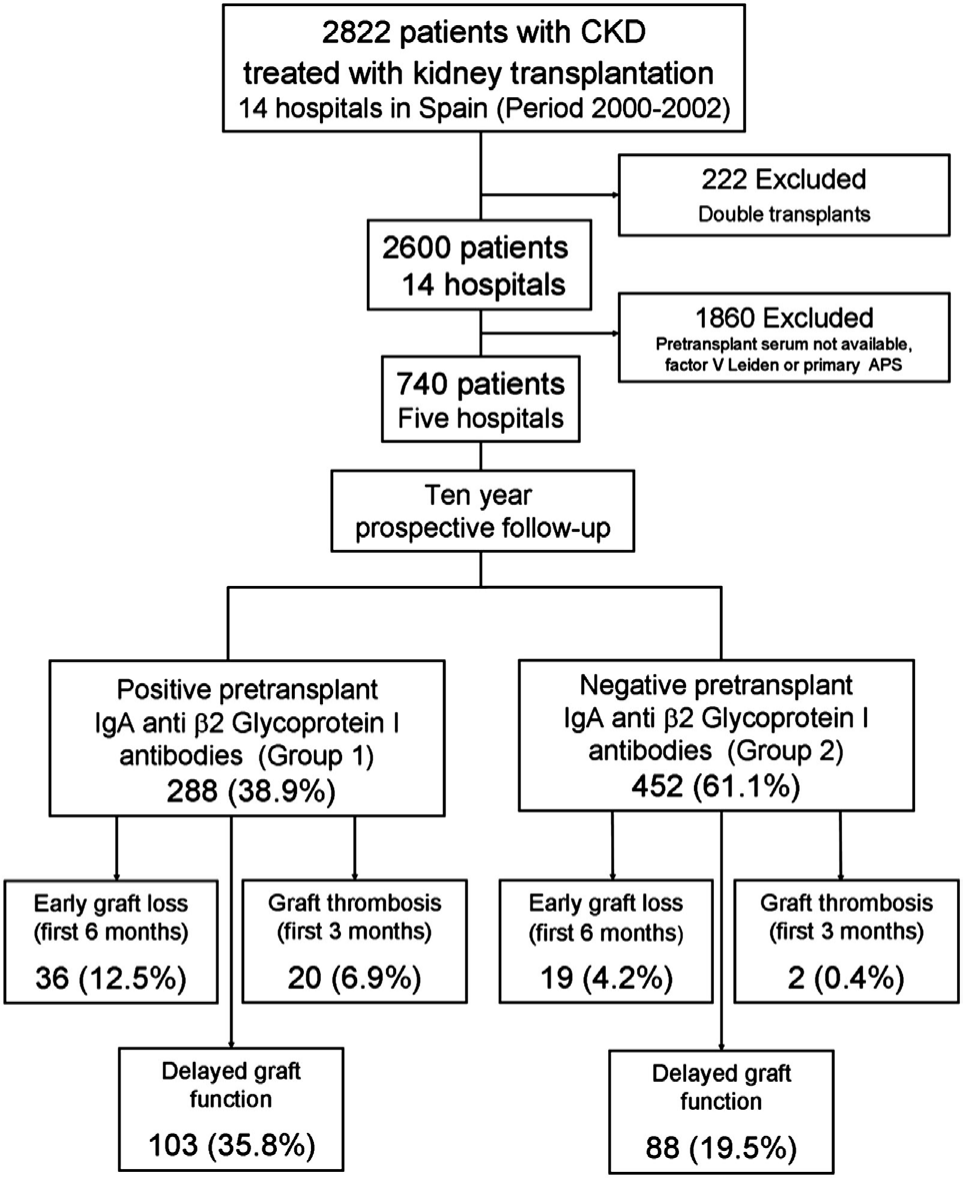
Disposition of study and early outcomes. This cohort included 740 patients from five renal transplant units that were followed-up for 10 years.

A total of 740 patients who had received a kidney transplant in a 3-year period (from 01/01/2000 to 12/31/2002) were evaluated in a 10-year prospective follow-up study. Presence of aPL was examined in pretransplant serum samples used for donor–recipient crossmatch (disposition algorithm and flow diagram, Figure 1).

The centers and number of patients in each center included in the study were as follows: Unit-1 (*N* = 269): “Hospital 12 de Octubre” (Madrid); Unit-2 (*N* = 86): “Hospital Ramon y Cajal” (Madrid); Unit-3 (*N* = 63): “Hospital Marques de Valdecilla” (Santander); Unit-4 (*N* = 282): “Hospital de Cruces” (Bilbao); and Unit-5 (*N* = 20): “Hospital la Paz” (Madrid).

#### Primary Aim

The primary aim is to confirm the prevalence of pretransplant IgA-aB2GP1 antibodies and its influence on outcomes after kidney transplantation in a multicenter study. Main endpoints: (A) early graft loss and (B) graft loss by thrombosis. Secondary endpoint: delayed graft function (DGF).

#### Secondary Aims

The secondary aims are to perform a Replication Study comparing the results from the hospitals 2 to 5 with the results from hospital 1 that had previously described such a prevalence. Secondary endpoints: prevalence of IgA-aB2GP1 antibodies, graft and patient survival, and causes of graft loss and death.

#### Ethical Issues

The “Forum renal” study (prospective observational and non-interventional) was approved by all the Ethics Committees and Nephrology Departments of the 14 hospitals, assuring data confidentiality (15, 16). The patients were not asked to sign an informed consent because the Spanish legislation does not require it for observational studies without intervention. This study was approved by the ethics committee of the five hospitals, assuring data confidentiality. To control the center effect, each participating hospital was assigned a blinded code.

In addition, prior to centralizing the laboratory and database work, the study was submitted for approval to the Hospital 12 de Octubre Ethics Committee for Clinical Research and it received a favorable report (Reference Number CEIC-14/021).

### Patients

Seven hundred forty patients (99.1% Caucasian) received a kidney transplant from heart-beating (brain dead) donors. Although an exhaustive coagulation study was not performed, all patients were negative for Factor V Leiden (disposition algorithm and outcomes, Figure 1). No patient was diagnosed of hemolytic uremic syndrome as an original disease. All of them were complement-dependent cytotoxicity crossmatch negative. All 740 patients were followed-up for a period of up to 10 years or until graft failure or death.

### Database

The donor and recipient characteristics were stored in an anonymized database. The pretransplant variables included were gender, age, original disease, serology, immunological data, time on dialysis, and pretransplant conditions. In this way, hypertension, hyperlipidemia, diabetes, body mass index (BMI), smoking, and pretransplant cardiovascular disease were recorded. Immunosuppressive drug treatment was also included.

After surgery, incidence of thrombotic events, cardiovascular events, neoplasia, DGF, and acute rejection (AR) episodes were recorded. Patient and graft survival (GS), and causes of mortality and graft loss were also recorded.

### Immunosuppressive Treatment

The most frequently used immunosuppressive protocol (88.5% of patients) was based on triple therapy with calcineurin inhibitors, mainly tacrolimus, associated with steroids and MMF with or without induction. In patients older than 60 years who received kidneys from donors older than 60 years, immunosuppression regimen was based on Cyclosporine (CsA), steroids, and MMF with or without induction. Immunosuppressive treatments in all the patients are described in Table S1 in Supplementary Material.

### Definitions

#### Thrombotic Events

Thrombotic events were defined as venous thrombosis, arterial thrombosis, graft thrombosis, pulmonary thromboembolism acute stroke, or transient ischemic attack. Thrombotic episodes were diagnosed by imaging techniques or by histological study (9).

#### Graft Thrombosis

Graft thrombosis was diagnosed with imaging techniques. Furthermore, most of the patients who suffered graft thrombosis underwent a transplactectomy. Immediately after the surgery, the kidneys were studied in the Pathology Department, the results of macroscopic and histopathology analysis confirmed the presence of graft thrombosis.

#### Graft Loss

Death of the patient or loss of kidney function that requires the beginning of permanent renal replacement therapy. It is considered early graft loss if it occurs in the first 6 months after the transplant. Censored graft loss is graft loss excluding death of the patient.

#### Delayed Graft Function

Delayed graft function is a form of posttransplantation acute renal failure defined as a temporary graft non*-*function that requires hemodialysis during the first week after surgery. DFG was diagnosed once discarded hyperacute rejection, vascular complications, and urinary tract obstruction.

#### Arterial Hypertension

Arterial hypertension was defined as blood pressure greater than 140/90 mm Hg.

#### Primary Non-Function

Primary non-function was considered to exist in grafts with good perfusion that never functioned in which a biopsy study had excluded other causes of graft dysfunction as AR.

#### Cardiovascular Event

Cardiovascular event was considered if at least one of the following was present: heart failure, angina, coronary revascularization, myocardial infarction, stroke, or peripheral bypass.

#### Acute Rejection

Acute rejection was applied to acute deterioration in allograft function associated with specific histopathologic changes in the graft.

#### Clinically Suspected AR

Clinically suspected AR is defined as patients with AR clinical criteria lacking histological data that confirmed the diagnostic.

#### Panel Reactive Antibody Score (PRA)

Panel Reactive Antibody Score was defined as the percentage of the general population to which the patient reacts by preformed antibodies. PRA was studied by complement-mediated cytotoxicity using pooled lymphocyte panel with at least 35 unrelated genotypes. Patients were considered as “sensitized” with PRA values ≥50%.

#### Hyperlipidemia

Hyperlipidemia was defined as when hypertriglyceridemia (>150 mg/dL) or hypercholesterolemia (>200 mg/dL) were seen.

#### Diabetes Mellitus

Diabetes mellitus was diagnosed in patients with fasting plasma glucose greater than 126 mg/dL (7.0 mmol*/*L).

#### Normal Weight

Normal weight was defined by a BMI range from 18.5 to 25 kg/m^2^. Overweight was defined when BMI is >30 kg/m^2^.

### Laboratory Determinations

Autoantibodies were measured in pretransplant serum used for crossmatch or in a serum sample obtained up to 15 days before transplantation. All the aPL determinations were performed in center 1.

IgA-aB2GP1 antibodies were quantified in all the centers by enzyme-linked immunosorbent assay (ELISA) using the QUANTA Lite B2 GPI IgA (INOVA Diagnostics Inc., San Diego, CA, USA). A unique assay lot was used for the analysis of the samples from centers 2, 3, 4, and 5.

The anti-cardiolipin (aCL) and aBGPI antibodies of IgG and IgM isotypes in patients from center 1 were measured with QUANTA Lite aCL IgG, QUANTA Lite B2 GPI IgG, QUANTA Lite aCL IgM, and QUANTA Lite B2 GPI IgM (INOVA Diagnostics Inc.). In patients from centers 2, 3, 4 and 5, these were measured using BioPLex 2200 multiplex immunoassay system APLS IgG and IgM (Bio-Rad, Hercules CA, USA).

Antibody levels higher than 18 U/mL were considered positive for aPL of IgG and IgM isotypes and higher than 20 U/mL were considered positive for IgA-aB2GP1. The cutoff values were those recommended by the manufacturer, which coincided with those determined in the healthy population in our country (17, 18).

### Statistical Methods

Results were expressed as mean ± SE, or absolute frequency and percentage. Association between qualitative variables was determined with Pearson’s Chi-square test or Fisher’s exact test, when appropriate. Comparisons were performed using the Mann–Whitney *U* test in scaled variables with two categories. Probabilities less than 0.05 were considered significant.

Survival was calculated using the Kaplan–Meier Method and differences between the distributions of survival were assessed with the log-rank test.

Multivariate analyzes of graft loss and graft thrombosis-associated variables were performed using Cox regression (proportional hazards model). The relative measure of an effect was expressed as hazard ratio (HR).

Multivariate analysis of DGF (dichotomous outcome concentrated in a very short period of time) was performed using logistic regression model (19). Probabilities less than 0.05 were considered significant.

The policy regarding donor–recipient selection was based on trying to match recipients and donors with similar ages. Therefore, donor age is a recipient age-dependent variable. Donor age was not included as a statistical analysis variable except when studying DGF because it is more associated with donor age than recipient age in the literature (20).

Data were processed and analyzed using Medcalc for Windows version 16.1 (MedCalc Software, Ostend, Belgium).

## Results

### Antiphospholipid Antibodies

The average pretransplant levels of aCL antibodies were IgM 5.4 U/mL ± 0.7 and IgG 4.0 U/mL ± 0.4. Mean levels of aB2GP1 antibodies were as follows: IgM 4.3 U/mL ± 0.8, IgG were 4.1 U/mL ± 0.5, and IgA were 32.4 U/mL ± 1.8 (Table S2 in Supplementary Material). Patients whose antibody levels were above the cutoff were considered positive. Prevalence of aCL positive patients was 1.1% for IgM and 1.2% for IgG. Prevalence of aB2GP1 antibodies patients was 1.6% for IgM and 1.2% for IgG.

### Patients with IgA-aB2GP1 Antibodies

Two hundred eighty-eight (38.9%) patients were positive for IgA-aB2GP1 antibodies (Group-1) and 452 were negative (Group-2). Patients in Group-1, were immunologically less complex and there were fewer retransplanted patients (10.8 vs. 17.5%; *p* = 0.017) and less hyperimmunized patients (6.6 vs. 11.9%; *p* = 0.024). The prevalence of dyslipidemia and hypertension was slightly higher in Group-1. The remaining pretransplant characteristics did not differ between both groups (Table 1). The correlation between recipient age and IgA-aB2GP1 levels was very weak (Correlation coefficient *r* = 0.184, 95% CI: 0.114–0.253).

**Table 1:** Pretransplant condition of patients in Group-1 (positive for IgA-aB2GP1 antibodies) and in Group-2 (negative patients).

	Group-1 (*N* = 288)		Group-2 (*N* = 452)		
			
Condition	Patients/mean	%/SE	Patents/mean	%/SE	*P*-value
Sex (women)	107	37.2%	198	43.8%	N.S.
Age (years)^a^	51.9	±0.8	47.4	±0.6	<0.001
Donor age (years)^a^	47.9	±1	44.2	±0.8	N.S.
Body mass index^a^	25.5	±0.3	24.9	±0.2	N.S.
Time on dialysis (months)^a^	36.5	±2.2	28.8	±2.0	N.S.

Type of dialysis					
Hemodialysis	217	75.3%	342	75.7%	N.S.
Peritoneal dialysis	58	20.1%	100	22.1%	N.S.
Both	12	4.2%	8	1.8%	N.S.
Undialyzed	1	0.3%	2	0.4%	N.S.
Panel reactive antibody score (PRA) at transplantation >50%	5	1.7%	19	4.2%	N.S.
Historical PRA >50%	19	6.6%	54	11.9%	0.024
Previous kidney transplant	31	10.8%	79	17.5%	0.017
Cold ischemia (h)^a^	19.5	±0.3	19.8	±0.3	N.S.

Associated conditions Diabetes mellitus				
Type 1 diabetes	14	4.9%	17	3.8%	N.S.
Type 2 diabetes	22	7.6%	24	5.3%	N.S.
Dyslipidemia	90	31.2%	98	21.7%	0.004
Hypertension	230	79.9%	311	68.8%	0.001

Causes CKD					
Chronic glomerulonephritis	73	25.3	137	30.3%	N.S.
Interstitial kidney disease	41	14.2%	59	13.1%	N.S.
Nephroangiosclerosis	20	6.9%	40	8.8%	N.S.
Polycystic kidney disease	47	16.3%	71	15.7%	N.S.
Diabetes mellitus	27	9.4%	29	6.4%	N.S.
Unknown	45	15.6%	67	14.8%	N.S.
Others	35	12.2%	49	10.8%	N.S.

*N.S., non-significant*.

*^a^Mann–Whitney test was used because variable is not normally distributed*.

### Clinical Events and Course in the Early Posttransplant Period (6 Months)

Thirty-six patients in Group-1 (12.5%) lost their graft during the first semester after transplantation vs. 19 in the Group-2 (4.2%, *p* < 0.001). At 3 months, the percentage of patients with graft loss in the Group-1 was also significantly higher than in Group-2 (10.8 vs. 2.9%, *p* < 0.001) (Table 2). Differences between patients with early graft loss (<6 months) and remaining patients were age (55.7 ± 1.7 vs. 48.6 ± 0.5 years, *p* < 0.001), presence of DGF (50.9 vs. 23.8%, *p* < 0.001), positivity for IgA-aB2GP1 antibodies (65.5 vs. 36.8%, *p* < 0.001), and a higher proportion of patients with nephroangiosclerosis as cause of end-stage renal disease (ESRD) (20 vs. 7.2%, *p* = 0.001) (Table 3). As the risk of graft loss and graft thrombosis is partially dependent on the donor factors, we performed an analysis of same-donor paired kidneys (21) showing the same results (data not shown).

**Table 2:** Posttransplant events of patients in Group-1 (positive for IgA-aB2GP1 antibodies) and in Group-2 (negative patients).

	Group-1 (*N* = 288)		Group-2 (*N* = 452)		
			
Condition	Patients/mean	%/SE	Patents/mean	%/SE	*P*-value
Delayed graft function (DGF)	103	35.8%	88	19.5%	<0.001
Graft loss on the complete follow-up (global 29.6%)	114	39.6%	105	23.2%	<0.001
First-month (global 3.9%)	20	6.9%	9	2%	0.001
First-trimester (global 5.9%)	31	10.8%	13	2.9%	<0.001
First semester (global 7.4%)	36	12.5%	19	4.2%	<0.001
First year (global 8.5%)	38	13.2%	25	5.5%	<0.001

Causes graft loss first semester					
Acute rejection (AR)	7	2.4%	1	0.2%	0.014
Non-functioning graft	1	0.3%	6	1.3%	N.S.
Death (with a functioning kidney)	4	1.4%	4	0.9%	N.S.
Cardiovascular diseases (CVDs)	1		0		N.S.
Infections	3		0		N.S.
Sudden death	0		2		N.S.
Others	0		2		N.S.
Graft thrombosis	20	6.9%	2	0.4%	<0.001
Others	4	1.4%	6	1.3%	N.S.
Graft loss from month 7 to end of follow-up	78	27.1%	86	19%	<0.001
AR	5	1.7%	0	0%	0.019
Death (with a functioning kidney)	41	14.2%	47	10.4%	N.S.
CVDs	9		11		N.S.
Infections	8		7		N.S.
Cancer	6		10		N.S.
Sudden death	3		3		N.S.
Others	15		16		N.S.
Chronic allograft nephropathy	26	9%	32	7.1%	N.S.
Others	6	2.1%	7	1.5%	N.S.

Cardiovascular events					
Myocardial infarction	7	2.4%	18	4%	N.S.
Stroke	18	6.3%	9	2%	0.005
Angina pectoris	9	3.1%	7	1.5%	N.S.
Patients with AR episodes	28	9.7%	43	9.5%	N.S.
Death in follow-up	57	19.8%	55	12.2%	0.005
Death first semester	11	3.8%	8	1.8%	N.S.
Death from months 7 to 24	46	16%	47	10.4%	0.034

*N.S., non-significant*.

**Table 3:** Clinical characteristics of patients with early graft loss vs. patients with functioning graft at 6 months posttransplant.

Condition	Early graft loss (*N* = 55)		Functioning graft (*N* = 685)		
			
	*N*/mean	%/SE	*N*/mean	%/SE	*P*
Sex (women)	17	30.9%	288	42%	N.S.
**Age (years)^a^**	55.7	±1.7	48.6	±0.5	**<0.001**
Donor age (years)^a^	55.2	±2.2	44.9	±0.7	<0.001
Body mass index^a^	25.6	±0.6	25.1	±0.2	N.S.
Time on dialysis (months)^a^	44.9	±7.1	32.6	±1.6	N.S.
Pretransplant clinical characteristics Diabetes mellitus	5	9.1%	72	10.5%	N.S.
Type 1 diabetes	2	3.6%	29	4.2%	N.S.
Type 2 diabetes	3	5.5%	43	6.3%	N.S.
Dyslipidemia	18	32.7%	170	24.8%	N.S.
Hypertension	44	80%	497	72.6%	N.S.
**Patients IgA-aB2GP1 positive**	36	65.5%	252	36.8%	**<0.001**
Causes CKD					
Chronic glomerulonephritis	15	27.3%	195	28.5%	N.S.
Interstitial kidney disease	9	16.4%	91	13.3%	N.S.
**Nephroangiosclerosis**	**11**	**20%**	**49**	**7.2%**	**0.002**
Polycystic kidney disease	6	10.9%	112	16.4%	N.S.
Diabetes mellitus	3		53		N.S.
Unknown	4		108		N.S.
Others	7		77		N.S.
Transplant-associated factors					
Previous kidney transplant	13		97		N.S.
Panel reactive antibody score (PRA) at time of transplant >50%	4	7.3%	20	2.9%	N.S.
Historical PRA >50%	7		66		N.S.
Cold ischemia (h)^a^	20.7	±0.6	19.6	±0.2	N.S.
**DGF**	**28**	**50.9%**	**163**		**<0.001**

*N.S., non-significant*.

*The variables that were selected for the multivariate analysis are marked in bold*.

*^a^Mann–Whitney test was used because variable is not normally distributed*.

**Figure 2:**
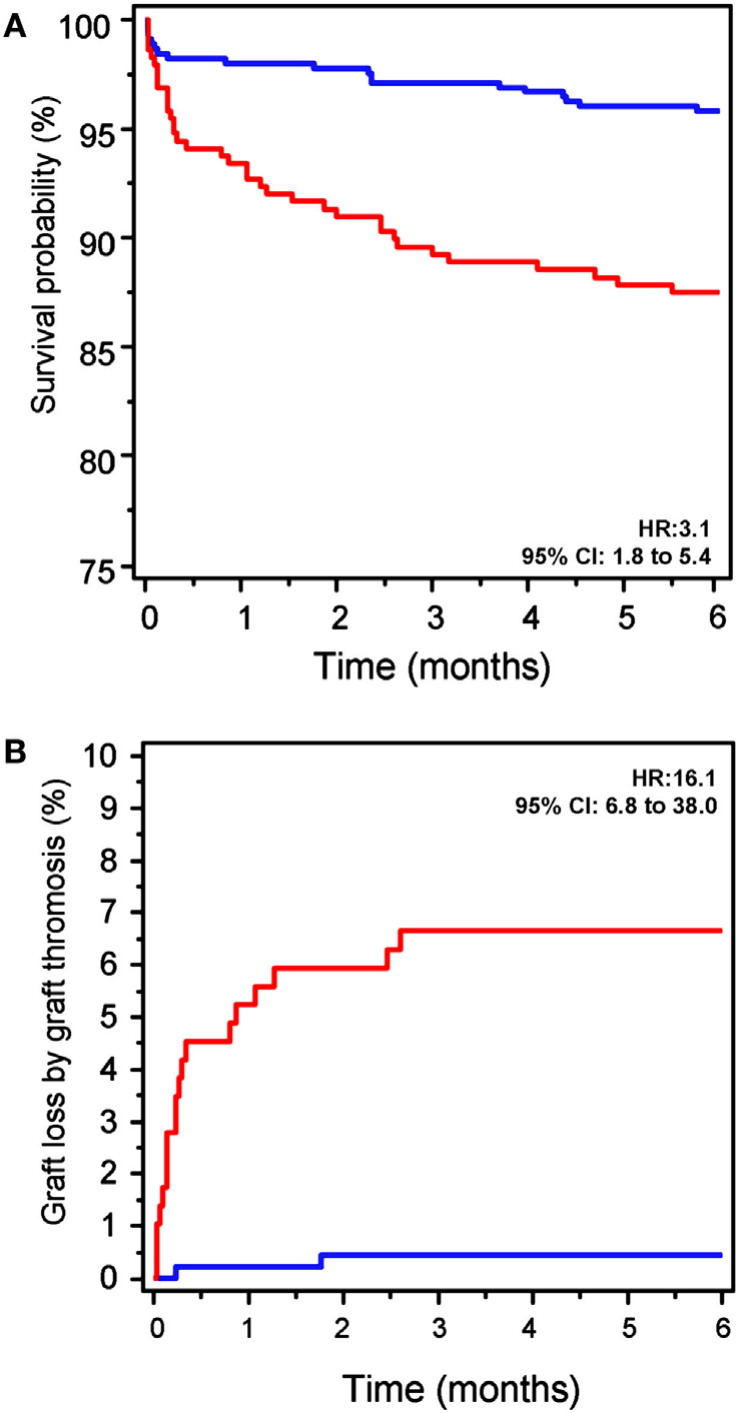
Early graft loss. **(A)** Evolution of graft survival (GS) at the first 6 months of the follow-up. GS in patients in Group-1 (positive for IgA-aB2GP1 antibodies, red line) was significantly lower than that observed in Group-2 (blue line). HR, hazard ratio (Kaplan–Meier analysis). **(B)** Graft loss by thrombosis in the 6 months after transplantation was significantly higher in patients in Group-1 (red) than in patients in Group-2 (blue).

A Kaplan–Meier survival analysis showed significantly lower 6-month GS rates in Group-1 (HR: 3.10, 95% CI: 180–5.35, *p* < 0.001, Figure 2A). Graft thrombosis was the most common cause of graft loss (22 patients, 61% of losses), this occurring more frequently in Group-1 (6.9 vs. 0.4%, *p* < 0.001, Figure 2B).

Delayed graft function was also significantly higher in Group-1 (35.8 vs. 19.5%; *p* < 0.001). There were no differences regarding AR episodes in both groups (Table 2).

### IgA anti-B2GP1 Antibodies Are an Independent Risk Factor for Early Graft Loss

Early graft loss-associated factors that were significant in the univariate analysis (Table 3) were included in a multivariate analysis [Table 4 **(A)**].

**Table 4:** Multivariate analysis.

Factors	Univariate			Multivariate		
		
	Hazard ratio (HR)	95% CI	*P*-value	HR	95% CI	*P*-value
**A. Early graft loss**
Patients IgA-aB2GP1 positive	3.11	1.78–5.41	<0.001	**2.49**	**1.40–4.43**	**0.002**
Recipient age (years)	1.04	1.02–1.06	<0.001	**1.02**	**1.00–1.05**	**0.042**
Nephroangiosclerosis	3.01	1.55–5.82	0.001	**2.61**	**1.32–5.17**	**0.006**
Delayed graft function (DGF)	3.14	1.85–5.33	<0.001	**2.35**	**1.37–4.04**	**0.002**

**B. Early graft loss by thrombosis**
IgA-aB2GP1 positive	16.09	3.76–68.85	<0.001	**13.83**	**3.17–60.27**	**<0.001**
DGF	4.29	1.83–10.03	<0.001	2.42	1.01–5.81	0.047
Cold ischemia time (h)	1.09	1.01–1.17	0.024	**1.09**	**1.056–3.233**	**0.041**

	**Odds ratio (OR)**	**95% CI**	*****P***-value**	**OR**	**95% CI**	*****P***-value**

**C. DGF**
IgA-aB2GP1 positive	2.30	1.65–3.22	<0.001	**2.08**	**1.47–2.95**	**<0.001**
Donor age (years)	1.02	1.01–1.03	<0.001	**1.02**	**1.01–1.03**	**0.003**
Cold ischemia (h)	1.04	1.01–1.07	0.011	1.03	1.00–1.07	0.050
Body index mass	1.07	1.04–1.11	<0.001	**1.06**	**1.02–1.10**	**0.002**
Hypertension	1.67	1.12–2.49	0.012	**1.56**	**1.03–2.37**	**0.039**
Time on dialysis (months)	1.00	1.00–1.01	0.307	–	–	–

*(A) Cox proportional regression multivariate analysis (*p* < 0.001) of graft loss-associated variables significant in univariate analysis. (B) Cox proportional regression multivariate analysis (*p* < 0.001) of variables associated with graft loss by thrombosis. (C) Logistic regression multivariate analysis (*p* < 0.001) of DGF-associated variables significant in univariate analysis*.

*The variables that were selected for the multivariate analysis are marked in bold*.

Presence of IgA-aB2GP1 antibodies continued to be an independent and significant risk factor for graft loss after adjusting for other risk factors (HR: 2.49; 95% CI: 1.40–4.43, *p* = 0.002). Recipient age, presence of DGF, and nephroangiosclerosis as cause of ESRD were also independent risk factors for early graft loss [Table 4 (A)].

### IgA-aB2GP1 Antibodies Are an Independent Risk Factor for Graft Thrombosis

IgA-aB2GP1, cold ischemia time, and age were identified in univariate analysis as significant and associated factors for graft thrombosis (Table S3 in Supplementary Material). In a Cox proportional regression multivariate analysis, cold ischemia time, DGF, and especially IgA-aB2GP1-ab (HR: 13.83; 95% CI: 3.17–60.27; *p* < 0.001) were identified as independent risk factors for graft thrombosis [Table 4 (B)].

No significant differences were observed between the group I patients who suffered graft loss due to thrombosis and the remaining patients of this same group who had cardiovascular risk factors: dyslipidemia (5.6 vs. 7.6%; *p* = 0.533), hypertension (6.1% vs. 10.3%; *p* = 0.255), type 2 diabetes (9.1 vs. 6.8%; *p* = 0.681), and BMI (23.9 ± 0.8 vs. 25.6 0.3; *p* = 0.128).

### IgA-aB2GP1 Antibodies Are an Independent Risk Factor for DGF

Variables previously significantly associated to DGF (Table S4 in Supplementary Material) were analyzed in a logistic regression univariate analysis. Those that continued to be significant were studied in a multivariate analysis: donor age, BMI, hypertension, and especially IgA-aB2GP1 antibodies [odds ratio (OR): 2.08, 95% CI: 1.47–2.95; *p* < 0.001] were identified as independent risk factors for DGF [Table 4 (C)].

### Late Posttransplant Period (from 7 Months to 10 Years)

**Figure 3:**
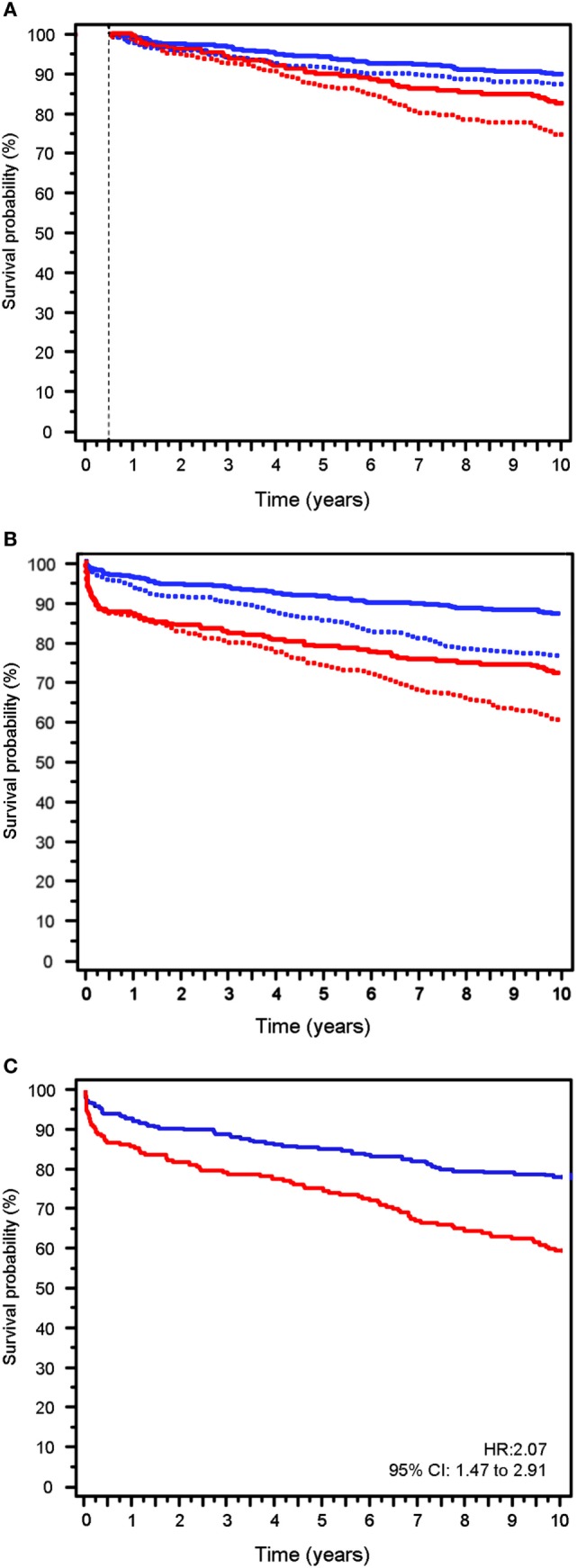
Graft survival (GS) on the complete follow-up. **(A)** GS from the end of the sixth month to the end of follow-up. Death-censored GS (solid line) vs. uncensored GS (dotted line). **(B)** GS during the 10 years of follow-up. Death-censored GS (solid line) vs. uncensored GS (dotted line). **(C)** Graft loss in the follow-up excluding Center 1 patients (uncensored data). Red lines patients in Group-1. Blue lines patients in Group-2.

Graft survival (Figure 3) was significantly lower (Kaplan–Meier analysis) in Group-1, both non-censored GS (HR: 1.63; 95% CI: 1.19–2.25; *p* = 0.002) and death-censored GS (HR: 1.80; 95% CI: 1.12–2.89; *p* = 0.009).

Causes of graft loss in this period were similar in both groups except for death with a functioning kidney that was more frequent in Group-1 patients (OR: 1.60; 95% CI: 1.02–2.51; *p* = 0.042).

### Complete Follow-up (0–120 Months)

Non-censored GS was 70.4% at 10 years when the total group was considered. GS analysis (Kaplan–Meier) showed that graft loss was higher in Group-1 vs. Group-2: survival was 60.4 vs. 76.8%; HR: 1.91; 95% CI: 1.45–2.52; *p* < 0.001 (Figure 3B, dotted lines).

Death-censored GS (Kaplan–Meier analysis) was lower in Group-1 than Group-2: 76.1 vs. 86.5%; HR: 2.34; 95% CI: 1.61–3.39; *p* < 0.001 (Figure 3B, solid lines).

**Figure 4:**
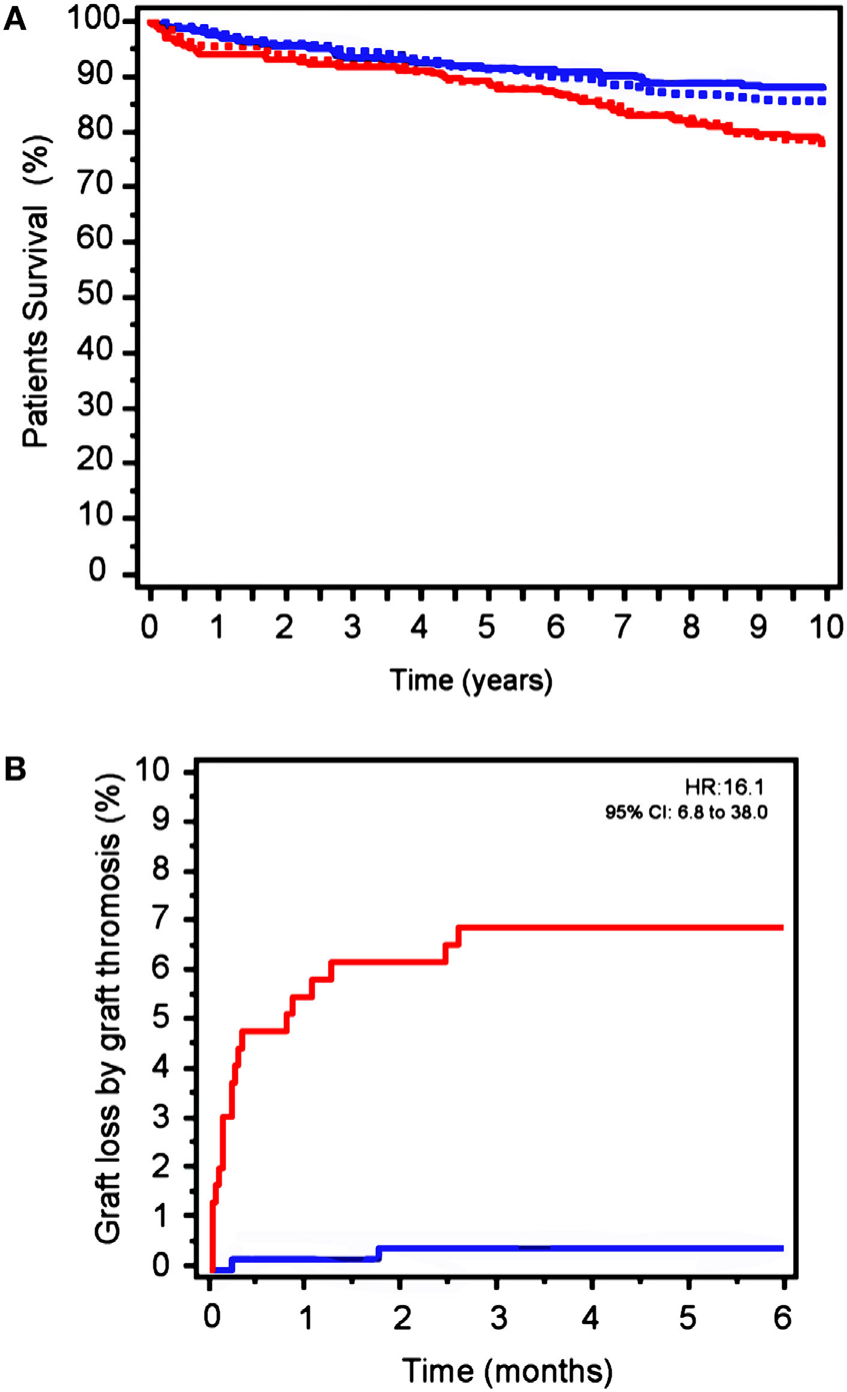
**(A)** Mortality in patients in Group-1 (red) vs. Group-2 (blue) in the follow-up. Solid lines: excluding center 1. Dotted lines: complete study. **(B)** Graft-loss by graft thrombosis excluding center 1. Observation: the survival curves from the study that excludes center 1 and the survival curves from the complete study are so similar that they practically overlap and impede a correct visualization.

Global mortality in the follow-up was 15.1%, this being significantly higher in Group-1 (19.8 vs. 12.2%, *p* = 0.005; Table 2). Therefore, survival probability was lower in Group-1 (HR: 1.53; 95% CI: 1.07–2.18; *p* = 0.015, Figure 4A).

### GS and Outcomes Excluding Center 1

When patients from centers 2, 3, 4, and 5 were considered alone with the exclusion of center 1, GS (Figure 3C) was also significantly lower in Group-1 vs. Group-2 patients, at 6 months (86.9 vs. 94.1%; HR: 2.30; 95% CI: 1.24–4.24; *p* = 0.007) and at 10 years (71.6 vs. 86.5%; HR 2.07 95% CI: 1.47–2.91; *p* < 0.001).

Notably, graft thrombosis (6 vs. 0.7%; OR: 8.66; 95% CI: 1.92–39.16; *p* = 0.005; Figure 4B) and DGF (39.7 vs. 22.4%; OR: 2.28; 95% CI: 1.52–3.40; *p* < 0.001) were significantly more frequent in patients in Group-1 than observed in Group-2 patients.

Remarkably, in a Cox proportional regression multivariate analysis (Table S5 in Supplementary Material), IgA-aB2GP1 antibodies continued to be an independent and significant risk factor associated with early graft-loss (HR: 1.90; 95% CI: 1.00–3.61; *p* = 0.049) and graft thrombosis (HR: 8.28; 95% CI: 1.75–39.07; *p* = 0.008). Likewise, IgA-aB2GP1 continued to be an independent risk factor for DGF in a multivariate logistic regression analysis (OR: 2.19; 95% CI: 1.43–3.37; *p* < 0.001).

Mortality was also higher on Group-1 (21.6 vs. 12.5%; *p* = 0.008; HR: 1.81; 95% CI 1.15–2.85; *p* = 0.009).

## Discussion

We previously described that the presence of pretransplant IgA-aB2GP1 is an independent risk factor for early graft loss in two unicenter studies (7, 14) and graft thrombosis (14) after renal transplantation. For the first time, herein, we have been able to demonstrate in an observational, multicenter, and prospective study including 740 renal transplant patients from five hospitals of Spain that preformed IgA-aB2GP1 is an independent risk factor for early graft thrombosis. Therefore, the presence of pretransplant IgA-aB2GP1 may be considered as a new tool to predict early graft loss by thrombosis after renal transplantation. In addition, these important results can be the rationale to investigate if prophylactic treatment of pretransplant IgA-aB2GP1 positive patients could improve this catastrophic early complication.

In this study we found that 55 (7.4%) of renal transplant patients suffered early loss in the first 6 posttransplant months and 22 of them (40%) due to graft thrombosis. Therefore, graft thrombosis was the main cause of early graft loss, representing 3% of the total group, a percentage almost similar to the other series (22). In addition, it is important to consider that these findings were obtained from a database representing the clinical practice in our country, including all transplanted patients from brain-death donors during 2000–2002, without exclusions. Notably, 90% of patients with graft thrombosis exhibited pretransplant IgA-aB2GP1 and consequently this parameter was the most important significant risk factor for graft thrombosis. This interesting finding was also corroborated when only centers 2, 3, 4, and 5 were analyzed. This partial analysis that excluded center 1 was done in order to demonstrate that the data from center 1 were not conditioning the final results. Therefore, the relationship between renal graft thrombosis and the presence of pretransplant IgA-aB2GP1 seems to be very clearly significant.

The prevalence of anti-B2GP1 IgA was significantly lower in retransplanted patients than in those receiving a first transplant. This finding confirms that observed in previous studies, that is, that the prevalence of these autoantibodies is lower in patients who have undergone immunosuppressant treatment prior to transplantation, either due to an autoimmune illness or a previous transplant (11, 14).

The prevalence of hyperlipidemia and hypertension in Group-1 patients is higher than in those in Group-2. We could hypothesize that this higher prevalence could be related to their older age; however, there is no known explanation for this finding. The presence of these factors could affect the development of vascular conditions such as arteriosclerosis. However, as the prevalence of hyperlipidemia and hypertension does not show significant differences in the group I patients who have lost the graft due to thrombosis and the remaining group I patients who did not suffer graft loss, these factors do not seem to affect the vascular thrombosis of the graft.

Although it is known that the binding of antibodies with B2GP1 is critical to the development of events APS, the physiological functions of are unknown. Thus, the specific mechanism by which the antibodies act remains elusive (23).

Studies with electron microscopy suggest that the tridimensional structure of B2GP1 is not limited to a single conformation and it has been suggested that the geometry of the B2GP1 can alter their potential to interact with autoantibodies (24, 25).

Membrane-bound B2GP1 acquires a J-shaped structure and binding of anti-B2GP1 antibodies stabilizes the interaction of the protein with membrane phospholipids that is hypothesized to potentiate signaling through several receptors associated with prothrombotic cellular actions (26). Patients receiving a graft should undergo surgery, a known “second hit” to trigger the event. The risk of thrombotic events in carriers of IgA-aB2GP1 is higher for carriers of other thrombosis-associated risk factors such as smoking, infections, prolonged immobilization, use of estrogens, or surgical procedures (27). However, the contribution of these factors as second hits in association with IgA-aB2GP1 needs to be established in subsequent studies.

Since not all IgA-aB2GP1 positive patients develop thrombotic complications (18), the next step should be to find a marker that would identify the patients with a higher risk of thrombosis among those are IgA-aB2GP1 positive (28). We recently described that the presence of circulating immune complexes (CIC) of IgA bounded to B2GP1 was associated with occurrence of recent thrombotic events (29) and are a predictor of acute thrombotic events, including graft thrombosis after renal transplantation (30). However, we have not been able to detect the presence of CIC as, unfortunately, although the conditions of preservation of serum samples were adequate for the determination of IgA-aB2GP1, these conditions were not consistent with the maintenance of stable CIC and they had not been reliably determined in the present group of patients in some centers.

On the other hand, global results of this population at 10 years may be considered to be in agreement with other series of renal transplantation with deceased donors: patient survival 84.9%, DCGS and NCGS of 81 and 70.4%, respectively. It is important to note that our patients were closely followed-up in the renal transplant offices under the umbrella of a national health service with universal and lifelong support. These findings are in agreement with previous results in Spain (31). As we noted previously, IgAB2GP1 positive patients show lower survival figures than negative patients. Notably, the immunosuppressive protocol was based on calcineurin inhibitors and MMF. Only six patients did not receive calcineurin inhibitors as an initial immunosuppressive protocol, and therefore, we cannot discuss if m-TOR inhibitors can be useful in the prevention of vascular lesions associated with aPL (32). Furthermore, prior results have demonstrated a superior capacity of CNI over m-TOR inhibitors to inhibit alloantibodies production in renal transplantation (33, 34).

One of the main limitations is that these results were obtained during the first 3 years of the twenty-first century. Currently, donors and patients are older, show a high percentage of sensitized and retransplants as well as new forms of renal transplantation and with non-heart-beating donors. At present, and with these demographic changes, short- and long-term results could be different. At this point, such a limitation makes it necessary to design a long-term study.

Determination of IgA-aB2GPI antibodies in patients from all the centers was performed with the same diagnostic system (Quanta Lite ELISA). However, a different diagnostic system was used to quantify aPL of IgG and IgM isotype for center 1 (ELISA) than in rest of the centers (multiplex immunoassay). This change of methodology is irrelevant because the efficiency in determination of aPL of IgG and IgM isotypes using ELISA and multiplex diagnostic systems is very similar (35, 36). Furthermore, the average amount of antibody levels and the proportion of aPL of IgG and IgM isotype-positive patients was similar to those described in previous studies (7, 11).

The need to include the determination of aB2GP1 IgA in diagnostic protocols has been suggested recently. Currently, the IgA isotype is not included among the APS classification criteria of the APS, so that very few centers perform the IgA determination aB2GP1. For this reason, cases of IgA-mediated thrombosis are clearly underdiagnosed (37).

In summary, for the first time, we have been able to corroborate in a large cohort of patients from five hospitals that the presence of pretransplant IgA-aB2GP1 is an independent risk factor for graft thrombosis after renal transplantation, a devastating condition without available prevention and treatment. This finding can be the rationale for a prospective study to demonstrate if prophylactic anticoagulation can be useful to improve this early complication.

## Ethics Statement

The study was submitted to the Ethics Committee for Clinical Research (ECCR) of Hospital “12 de Octubre” and received a favorable report (Reference Number CEIC-14/021).

## Author Contributions

AS and JM conceived the project, designed the research, discussed the results, and wrote the manuscript. AS, MS, and JM-F performed the antiphospholipid determinations and were responsible for the database and the statistical analysis. JM, JG, MA, RM, FE, AA, NM, and EA were responsible for the patients’ care and clinical data collection. AS, MS, JM-F, JC, and ML-H were responsible for coordination of the Organ Transplant Waiting List Serum Bank. MM evaluated the histopathology. All authors contributed to the data interpretation, reviewed the manuscript, and agreed with the final version.

## Conflict of Interest Statement

The authors declare that the research was conducted in the absence of any commercial or financial relationships that could be construed as a potential conflict of interest. The reviewer KK and handling editor declared their shared affiliation.

## Abbreviations

OR, odds ratio; HR, hazard ratio; BMI, body mass index; PRA, panel reactive antibody score; aB2GP1, anti-beta-2 glycoprotein-I; aCL, anti-cardiolipin.
