# Supplementary material for: Pretransplant IgA-Anti-Beta 2 Glycoprotein I Antibodies As a Predictor of Early Graft Thrombosis after Renal Transplantation in the Clinical Practice: A Multicenter and Prospective Study
Source: Front Immunol. 2018 Mar 12;9:468. doi: 10.3389/fimmu.2018.00468 (PMC5857545; doi:10.3389/fimmu.2018.00468)
Supplement: Supplementary file 1 [file table_1.pdf]

***Supplementary Table 1**. Immunosuppressive treatment*

Immunosuppressive protocol	Number of patients
Calcineurin inhibitor + MMF + steroids	656
Cyclosporine A + MMF + steroids	*478*
Tacrolimus + MMF + steroids	*178*
Calcineurin inhibitor + steroids	67
Cyclosporine A + steroids	*38*
Tacrolimus + steroids	*29*
Others drug combinations	17
