# Supplementary material for: Pretransplant IgA-Anti-Beta 2 Glycoprotein I Antibodies As a Predictor of Early Graft Thrombosis after Renal Transplantation in the Clinical Practice: A Multicenter and Prospective Study
Source: Front Immunol. 2018 Mar 12;9:468. doi: 10.3389/fimmu.2018.00468 (PMC5857545; doi:10.3389/fimmu.2018.00468)
Supplement: Supplementary file 2 [file table_2.pdf]

Supplementary Table 2. *Antiphospholipid antibodies in the complete series of patients versus the subgroup that excludes patients from center 1. Non-significant differences were observed.*

Antibodies	All patients			Excluding Hospital 1		
	Mean	se	% positive	Mean	se	% positive
aB2GP1 IgA	32.4	±1.8	38.9%	34.5	±2.5	42.3%
aB2GP1 IgG	4.1	±0.5	1.2%	2.4	±0.4	1.2%
aB2GP1 IgM	4.3	±0.8	1.6%	2.2	±0.5	1.7%
aCL IgG	4	±0.4	1.2%	2.7	±0.4	1.2%
aCL IgM	5.4	±0.7	1.1%	2.4	±0.5	1.0%
