# Supplementary material for: Pretransplant IgA-Anti-Beta 2 Glycoprotein I Antibodies As a Predictor of Early Graft Thrombosis after Renal Transplantation in the Clinical Practice: A Multicenter and Prospective Study
Source: Front Immunol. 2018 Mar 12;9:468. doi: 10.3389/fimmu.2018.00468 (PMC5857545; doi:10.3389/fimmu.2018.00468)
Supplement: Supplementary file 3 [file table_3.pdf]

***Supplementary Table 3***

*Clinical characteristics of patients with graft loss by thrombosis versus patients without graft thrombosis. N.S.: Non-significant.* The variables that were selected for the multivariate analysis are marked in bold.*

| Condition. | Graft thrombosis | | No graft thrombosis | | |
|---|---|---|---|---|---|
| | N / mean | % / se | N / mean | % / se | p |
| Sex (women) | 9 | (40.9%) | 296 | (41.2%) | N.S. |
| Age (years) | 54. 2 | 2.8 | 49.0 | 0.5 | N.S. |
| Donor age (years) | 51.0 | 4.0 | 45.5 | 0.7 | N.S. |
| Body mass index | 24.1 | 0.74 | 25.2 | 0.2 | N.S. |
| Time on dialysis (months) | 33.0 | 8.1 | 33.6 | 1.6 | N.S. |
| Pretransplant Clinical characteristics | | | | | |
| Diabetes mellitus | 2 | (9.1%) | 75 | (10.4%) | N.S. |
| Type 1 Diabetes | 0 | (0%) | 31 | (4.3%) | N.S. |
| Type 2 Diabetes | 2 | (9.1%) | 44 | (6.1%) | N.S. |
| Dyslipidemia | 8 | (36.4%) | 350 | (48.7%) | N.S. |
| Hypertension | 16 | (72.7%) | 525 | (73.1%) | N.S. |
| **Patients IgA aB2GP1 positive** | 20 | (90.9%) | 268 | (37.3%) | **< 0.001** |
| Causes CKD | | | | | |
| Chronic glomerulonephritis | 10 | (45.5%) | 200 | (27.9%) | N.S. |
| Interstitial kidney disease | 4 | (18.2%) | 96 | (13.4%) | N.S. |
| Nephroangiosclerosis | 2 | (9.1%) | 58 | (8.1%) | N.S. |
| Polycystic kidney disease | 0 | (0%) | 118 | (16.4%) | 0.035 |
| Diabetes mellitus | 1 | (4.5%) | 55 | (7.7%) | N.S. |
| Unknown | 3 | (13.6%) | 109 | (15.2%) | N.S. |
| Other | 2 | (9.1%) | 82 | (11.4%) | N.S. |
| Transplant-associated factors | | | | | |
| Previous kidney transplant | 5 | (22.7%) | 105 | (14.6%) | N.S. |
| PRA at time of transplant >50% | 1 | (4.5%) | 23 | (3.2%) | N.S. |
| Historical PRA >50% | 3 | (13.6%) | 70 | (9.7%) | N.S. |
| **Cold ischemia (hours)** | 22.2 | ±0.8 | 19.6 | ±0.2 | **0.006** |
| **Delayed graft function** | 13 | (59.1%) | 178 | (24.8%) | **0.001** |
