# Supplementary material for: Pretransplant IgA-Anti-Beta 2 Glycoprotein I Antibodies As a Predictor of Early Graft Thrombosis after Renal Transplantation in the Clinical Practice: A Multicenter and Prospective Study
Source: Front Immunol. 2018 Mar 12;9:468. doi: 10.3389/fimmu.2018.00468 (PMC5857545; doi:10.3389/fimmu.2018.00468)
Supplement: Supplementary file 4 [file table_4.pdf]

Supplementary Table 4

Clinical characteristics of patients with delayed graft function. N.S.: Non-significant. The variables that were selected for the multivariate analysis are marked in bold.

Condition.	Delayed graft function		Rest of patients		
	N / mean	% / se	N / mean	% / se	p
Sex (women)	72	(37.7%)	233	(42.4%)	N.S.
**Age (years)**	52.5	±0.9	48	±0.6	<0.001
Donor age (years)	**50.5**	**±1.2**	**44**	**±0.8**	**<0.001**
**Time on dialysis (months)**	36.2	±2.8	32.6	±1.9	0.004
Body mass index	**26.2**	**±0.4**	**24.7**	**±0.2**	**<0.001**
Diabetes mellitus	40	(20.9%)	83	(15.1%)	N.S.
Type 1 Diabetes	6	(3.1%)	25	(4.6%)	N.S.
Type 2 Diabetes	17	(8.9%)	29	(5.3%)	N.S.
Dyslipidemia	95	(49.7%)	263	(47.9%)	N.S.
**Hypertension**	**153**	**(80.1%)**	**388**	**(70.7%)**	**0.015**
**Patients IgA aB2GP1 positive**	**103**	**(53.9%)**	**185**	**(33.7%)**	**<0.001**
Causes CKD					
Chronic glomerulonephritis	52	(27.2%)	158	(28.8%)	N.S.
Interstitial kidney disease	21	(11%)	79	(14.4%)	N.S.
Nephroangiosclerosis	21	(11%)	39	(7.1%)	N.S.
Polycystic kidney disease	28	(14.7%)	90	(16.4%)	N.S.
Diabetes mellitus	17	(8.9%)	39	(7.1%)	N.S.
Unknown	26	(13.6%)	86	(15.7%)	N.S.
Other	26	(13.6%)	58	(10.6%)	N.S.
Transplant-associated factors					
Previous kidney transplant	36	(18.8%)	74	(13.5%)	N.S.
PRA at time of transplant >50%	4	(2.1%)	20	(3.6%)	N.S.
Historical PRA >50%	18	(9.4%)	55	(10%)	N.S.
**Cold ischemia time (hours)**	**20.5**	**±0.4**	**19.3**	**±0.2**	**0.001**
