# Supplementary material for: Pretransplant IgA-Anti-Beta 2 Glycoprotein I Antibodies As a Predictor of Early Graft Thrombosis after Renal Transplantation in the Clinical Practice: A Multicenter and Prospective Study
Source: Front Immunol. 2018 Mar 12;9:468. doi: 10.3389/fimmu.2018.00468 (PMC5857545; doi:10.3389/fimmu.2018.00468)
Supplement: Supplementary file 5 [file table_5.pdf]

Supplementary Table 5. *Multivariate analysis excluding patients from center 1.*

(A) Cox proportional regression multivariate analysis (p<0.001) of early graft-loss associated variables significant in univariate analysis. (B) Cox proportional regression multivariate analysis (p<0.001) of variables associated to graft loss by thrombosis. (C) Logistic regression multivariate analysis (p<0.001) of delayed graft function-associated variables significant in univariate analysis.

FACTORS	UNIVARIATE			MULTIVARIATE		
**A. Early graft loss**	**HR**	**95% CI**	**P**	**HR**	**95% CI**	**P**
Patients IgA aB2GP1 positive	2.3	1.23 to 4.28	0.009	**1.90**	**1.00 to 3.61**	**0.049**
Recipient age (year)	1.03	1.01 to 1.06	0.006	1.02	1.00 to 1.05	0.102
Nephroangiosclerosis	3.83	1.83 to 8.01	<0.001	**2.97**	**1.39 to 6.37**	**0.005**
Delayed graft function	2.24	1.22 to 4.10	0.009	1.68	0.90 to 3.13	0.102
**B. Early graft loss by thrombosis**	**HR**	**95% CI**	**P**	**HR**	**95% CI**	**P**
IgA aB2GP1 positive	8.34	1.87 to 37.26	0.006	**8.28**	**1.75 to 39.07**	**0.008**
Delayed graft function	3.21	1.11 to 9.26	0.031	1.82	0.60 to 5.51	0.292
Cold ischemia time (hour)	1.07	0.99 to 1.17	0.086	**1.10**	**0.99 to 1.21**	**0.070**
**C. Delayed graft function**	**OR**	**95% CI**	**P**	**OR**	**95% CI**	**P**
IgA aB2GP1 positive	2.28	1.52 to 3.40	<0.001	**2.19**	**1.43 to 3.37**	**<0.001**
Donor age (year)	1.03	1.01 to 1.04	<0.001	**1.02**	**1.01 to 1.04**	**0.003**
Cold ischemia (hour)	1.02	1.01 to 1.04	<0.001	**1.05**	**1.01 to 1.09**	**0.010**
Body index mass	1.07	1.03 to 1.12	0.002	**1.06**	**1.01 to 1.11**	**0.020**
Hypertension	1.66	1.04 to 2.63	0.032	1.51	0.92 to 2.47	0.104
Time on dialysis (month)	1.00	0.998 to 1.01	0.322	--	--	--
